# Hematological Risk Factors for High-Altitude Headache in Chinese Men Following Acute Exposure at 3,700 m

**DOI:** 10.3389/fphys.2017.00801

**Published:** 2017-10-17

**Authors:** He Huang, Bao Liu, Gang Wu, Gang Xu, Bing-Da Sun, Yu-Qi Gao

**Affiliations:** ^1^Institute of Medicine and Hygienic Equipment for High Altitude Region, College of High Altitude Military Medicine, Third Military Medical University, Chongqing, China; ^2^Key Laboratory of High Altitude Environmental Medicine, Third Military Medical University, Ministry of Education, Chongqing, China; ^3^Key Laboratory of High Altitude Medicine, Chinese People's Liberation Army, Chongqing, China; ^4^The 12th Hospital of Chinese People's Liberation Army, Kashi Xinjiang, China

**Keywords:** headache, high-altitude headache, acute mountain sickness, hematological parameters, risk factor

## Abstract

**Background:** High-altitude headache (HAH) is a notably common disorder affecting the daily life of travelers ascending to high altitude. Hematological parameters are important clinical examinations for various diseases. Today, hematological characteristics of HAH remain unrevealed. Above all, we aimed to ascertain hematological characteristics and independent risk factors/predictors associated with HAH before and after exposure at 3,700 m.

**Methods:** Forty five healthy men were enrolled in present study. Demographic and clinical data, physiological and hematological parameters were collected 3 days before the ascent and after acute exposure at 3,700 m.

**Results:** HAH patients featured significantly lower white blood cell count (WBC), neutrophil count (NEU#) and percentage (NEU%), and higher percentage of lymphocyte (LYM%) at 3,700 m and significantly lower NEU#, reticulocyte count (RET#) and percentage (RET%) at sea level (all *P* < 0.05). HAH severity was significantly and negatively associated with WBC, NEU#, and NEU% at 3,700 m and RET# at sea level, whereas was positively associated with LYM% at 3,700 m (all *P* < 0.05). Moreover, we have found that RET# at sea level and NEU% at 3,700 m was an independent predictor and risk factor for HAH, respectively.

**Conclusion:** The present study is the first to examine the hematological characteristics of HAH. Furthermore, lower RET# at sea level and lower NEU% at 3,700 m is a novel independent predictor and risk factor for HAH, respectively.

## Introduction

Headache is the fundamental symptom for a diagnosis of acute mountain sickness (AMS) according to the Lake Louise Scoring System (Imray et al., 2010; Bartsch and Swenson, 2013; Davis and Hackett, 2017). In this context, high-altitude headache (HAH) is a defined and recognized headache disorder, which occurs within 24 h after ascending to an altitude of >2,500 m, based on the International Classification of Headache Disorders 3β criteria (Marmura and Hernandez, 2015; Kim et al., 2016). Approximately 80% of people are thought to experience headache after rapidly ascending to high altitudes, which is associated with disturbances in their daily life and work that create an important public health problem (Carod-Artal, 2014).

During recent decades, numerous researchers have systematically investigated the epidemiology, clinical manifestations, pathophysiological mechanisms, risk factors, prevention, and treatment of HAH (Wilson et al., 2009; Carod-Artal, 2014; Marmura and Hernandez, 2015). Clinical studies have demonstrated that HAH ordinarily presents as a sudden attack and partially mimics the features of migraine (Silber et al., 2003; Broessner et al., 2016). Current studies regarding the pathophysiological etiology of HAH have suggested that HAH can be induced by hypobaric hypoxia causing overperfusion of the microvascular beds, brain swelling, activation of the trigeminal vascular system, and sensitization of intracranial pain receptors (Burtscher et al., 2011). Furthermore, the known independent risk factors for HAH include increased heart rate (HR), posterior cerebral circulation, vertebral artery diastolic velocity, and anxiety; decreased pulse oxygen saturation (SpO_2_) and vigor; and a history of migraine or microRNA dysregulation (Silber et al., 2003; Lawley, 2011; Alizadeh et al., 2012; Bian et al., 2013, 2015; Liu et al., 2016; Dong et al., 2017; Guo et al., 2017). However, the actual underlying mechanisms, clinical manifestations and risk factors/predictors remain not entirely clear, despite accumulating evidence regarding HAH (Marmura and Hernandez, 2015).

Hematological parameters are important clinical markers and can be analyzed using simple, rapid, and inexpensive techniques (Devasena, 2017). Numerous researchers have suggested that hematological changes are closely associated with various diseases, including myelodysplastic syndrome, laryngeal squamous cell cancer, stable coronary artery disease, stroke, and acute pulmonary embolism (Nayak et al., 2011; List et al., 2012; Zorlu et al., 2012; Ayhan et al., 2013; Sahin et al., 2015; Soderholm et al., 2015; Hsueh et al., 2017). Moreover, recent studies have indicated that migraine conditions may be related to some hematological parameters, especially high hemoglobin levels and broad red blood cell distribution width (Celikbilek et al., 2013; Lippi et al., 2014). Thus, hematological changes are an attractive area for improving our understanding of HAH, although we are not aware of any studies that have examined this issue.

The present study was based on the hypothesis that hematological parameters would be closely related to HAH. Therefore, we evaluated the hematological characteristics of participants before and after exposure to high altitude, which may provide useful information regarding HAH-specific hematological phenotypes, independent risk factors, and predictors.

## Methods

### Participants

The inclusion criteria were healthy 18–60-year-old Chinese men whose primary residence was at an altitude of ≤1,000 m. The exclusion criteria were cardio-cerebrovascular diseases, respiratory diseases, neuropsychological diseases, kidney disorders, liver disorders, migraine or other headaches, history of travel to an altitude of >2,500 m during the last 2 years, and regularly drinking coffee or tea. Based on these criteria, we successfully recruited and enrolled 45 Chinese men who were healthy and 18–35 years old. The study's protocol was approved by the Third Military Medical University Ethics Committee (China), and complied with the requirements of the Declaration of Helsinki. All participants signed informed consent forms before their enrollment.

### Procedures

During the study, all participants rapidly ascended from Chongqing (sea level, altitude of 200 m) to Lhasa (altitude of 3,700 m) by train within 48 h. Three days before the ascent, the participants provided demographic data and underwent measurements of physiological and hematological parameters at sea level. Within 24 h after their arrival at 3,700 m, the participants underwent assessments of their physiological and hematological parameters, as well as HAH. During the study, the participants maintained the same diet (no coffee, tea, or alcoholic drinks) and avoided strenuous activity to ensure a similar level of physical activity. The participants were monitored by physicians for any signs of high-altitude cerebral or pulmonary edema (Hackett and Roach, 2004; Pennardt, 2013). The possibility of emergent cases of these conditions was addressed by planning for immediate evacuation and treatment using oxygen supplementation and medication.

### Demographic and clinical data collection

Three days before their ascent, the participants completed self-administered questionnaires to obtain their demographic data (body mass index [BMI], age, smoking history, and drinking history). After the acute exposure at 3,700 m, the physicians scored the participants based on the clinical features of HAH: headache (0 = none, 1 = mild, 2 = moderate, 3 = severe), the location of the headache (bilateral, frontal, frontal-temporal, or other), and factors that worsened the headache (exertion, movement, straining, coughing, or other). The diagnosis of HAH was based on fulfilling at least two of the International Classification of Headache Disorders 3β criteria (Marmura and Hernandez, 2015): mild or moderate severity; bilateral location; and aggravation caused by exertion, movement, straining, coughing, and/or bending.

### Measurement of physiological parameters

During the pre-exposure and post-exposure examinations, physicians measured the participants' physiological parameters, including HR, SpO_2_, diastolic blood pressure (DBP), and systolic blood pressure (SBP). The participants rested for 30 min before being evaluated using a sphygmomanometer (HEM-6200; OMRON, China) and a pulse oximeter (NONIN-9550; Nonin Onyx, USA).

### Blood collection and hematological parameter measurements

Pre-exposure and post-exposure venous blood samples were collected from the participants by qualified nurses using EDTA-coated tubes and standard procedures. The blood samples were stored at 4°C until the testing using the AU-2700 analyzers (Olympus, Tokyo, Japan) and commercial reagents. The hematological parameters were white blood cell count (WBC), red blood cell count (RBC), hematocrit (HCT), mean corpuscular volume, red cell distribution width and its coefficient of variation, platelet count, mean platelet volume, plateletcrit, platelet distribution width, platelet-large cell ratio, hemoglobin (HGB), mean corpuscular hemoglobin and concentration, neutrophil count (NEU#), reticulocyte count (RET#), lymphocyte count (LYM#), eosinophil count (EOS#), monocyte count (MON#), neutrophil percentage (NEU%), reticulocyte percentage (RET%), lymphocyte percentage (LYM%), eosinophil percentage (EOS%), and monocyte percentage (MON%) (Supplementary Table 1).

### Statistical analysis

**Figure 1:**
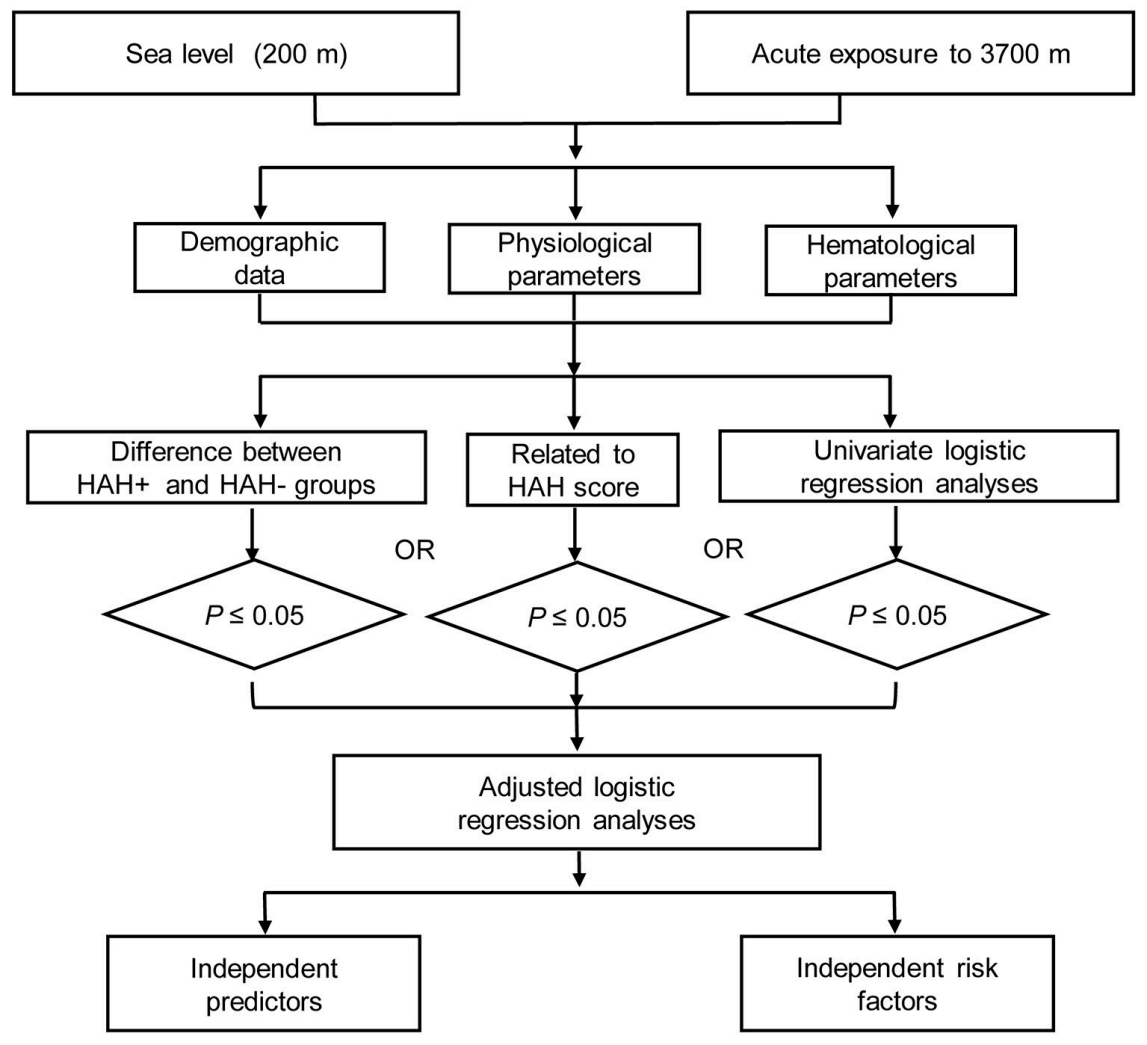
The flow diagram of this study. HAH+, participants with high-altitude headache (HAH); HAH−, participants without HAH.

The parameters' normality was assessed using the Shapiro-Wilk's test. Normally distributed data were presented as mean ± standard deviation, non-normally distributed data were presented as median (interquartile range), and categorical data were presented as number (percentage). Differences between the groups with and without HAH (HAH+/HAH−) were compared using the independent *t*-test for normally distributed data and the Mann-Whitney *U*-test for non-normally distributed data. Spearman correlation coefficients were evaluated for the relationships between the parameters and HAH severity scores. Univariate logistic regression was used to identify risk factors and predictors, and significant factors from the aforementioned analyses were included in adjusted logistic regression (Figure 1). All analyses were performed using IBM SPSS software (version 19; IBM Corp, Armonk, NY, USA), and differences were considered statistically significant at a *P*-value of < 0.05. All statistical methods and results were discussed with and guided by statisticians from the Third Military Medical University.

## Results

### Demographic and clinical characteristics

All 45 participants completed the pre-exposure and post-exposure assessments, and had complete data regarding their physiological and hematological parameters. The incidence of HAH was 51.1%, although no significant differences were observed between the HAH+ and HAH− groups for age, BMI, drinking, and smoking (all *P* > 0.05, Table 1).

**Table 1:** Differences of each variable between HAH+ and HAH− groups.

	**Measurements at sea level**		**Measurements at 3,700 m**	
	**HAH+ (23)**	**HAH− (22)**	**HAH+ (23)**	**HAH− (22)**
**DEMOGRAPHIC DATA**				
Age (year)	26.1 (3.0)	24.4 (9.0)	The same as sea level	
BMI (kg/m^2^)	20.8 (3.0)	22.2 (1.5)	The same as sea level	
Smoking	2 (8.70%)	3 (13.6%)	The same as sea level	
Drinking	7 (30.4%)	5 (22.7%)	The same as sea level	
**PHYSIOLOGICAL PARAMETERS**				
SBP (mmHg)	110.6 ± 8.2	110.1 ± 6.7	122.6 ± 13.7	123.3 ± 10.8
DBP (mmHg)	67.0 ± 7.4	67.2 ± 5.2	73.5 ± 10.3	71.7 ± 9.0
HR (beats/min)	65.3 ± 10.8	68.5 ± 8.4	83.3 ± 12.9	88.3 ± 8.8
SpO_2_ (%)	98.0 (1.0)	98.0 (1.0)	88.5 ± 2.6	87.2 ± 3.1
**HEMATOLOGICAL PARAMETERS**				
WBC (10^9^/L)	5.61 ± 1.18	6.31 ± 1.74	**6.33** ± **1.09**	**7.22** ± 1.62^*^
NEU^#^ (10^9^/L)	**2.94** ± **0.77**	**3.62** ± 1.35^*^	**3.26** ± **0.77**	**4.16** ± **1.31**^^#^^
LYM^#^ (10^9^/L)	2.23 ± 0.58	2.27 ± 0.56	2.53 ± 0.43	2.30 (0.42)
MON^#^ (10^9^/L)	0.25 (0.10)	0.28 (0.10)	0.51 ± 0.14	0.53 ± 0.13
EOS^#^ (10^9^/L)	0.10 (0.13)	0.14 ± 0.09	0.11 (0.09)	0.15 ± 0.09
NEU% (%)	52.13 ± 8.07	56.09 ± 8.00	**51.04** ± **6.75**	**56.70** ± **6.59**^^#^^
LYM% (%)	40.03 ± 7.95	36.75 ± 7.68	**37.70** ± **6.45**	**32.82** ± 6.13^*^
MON% (%)	4.67 ± 1.07	4.43 ± 1.01	8.10 ± 1.73	7.66 ± 1.14
EOS% (%)	2.70 (2.30)	2.70 (2.43)	1.90 (1.40)	2.07 ± 1.36
RBC (10^12^/L)	4.94 (0.46)	5.07 ± 0.37	5.41 (0.46)	5.51 ± 0.42
HGB (g/L)	149.00 (11.00)	150.09 ± 10.04	161.74 ± 11.04	165.95 ± 11.03
HCT (%)	45.03 ± 3.24	45.05 ± 3.05	48.09 ± 2.91	48.92 ± 2.91
RET^#^ (10^9^/L)	**37.93** ± **12.25**	**45.41** ± 9.52^*^	No data	
RET% (%)	**0.76** ± **0.25**	0.89 (0.22)^*^	No data	

*HAH+, participants with high-altitude headache (HAH); HAH−, participants without HAH; BMI, body mass index; SBP, systolic blood pressure; DBP, diastolic blood pressure; HR, heart rate; SpO_2_, pulse oxygen saturation*.

* P-value is 0.05 or less;

# *P-value is 0.01 or less; The bold data represent significant*.

### HAH-related parameters at sea level

Compared to the HAH− group, the HAH+ group had significantly lower pre-exposure values for RET% (0.76 ± 0.25% vs. 0.89 ± 0.22%), RET# (37.93 ± 12.25 10^9^/L vs. 45.41 ± 9.52 10^9^/L), and NEU# (2.94 ± 0.77 10^9^/L vs. 3.62 ± 1.35 10^9^/L) (all *P* < 0.05). There were no significant differences in the pre-exposure physiological parameters (all *P* > 0.05, Table 1, Supplementary Table 2A).

### HAH-related parameters at 3,700 m

Compared to the HAH− group, the HAH+ group had significantly lower post-exposure values for NEU# (51.04 ± 6.75 10^9^/L vs. 56.70 ± 6.59 10^9^/L), NEU% (3.26 ± 0.77% vs. 4.16 ± 1.31%), and WBC (6.33 ± 1.09 10^9^/L vs. 7.22 ± 1.62 10^9^/L), as well as significantly higher LYM% (37.70 ± 6.45% vs. 32.82 ± 6.13%) (all *P* < 0.05). Similar to the pre-exposure, there were no significant differences in the post-exposure physiological parameters (all *P* > 0.05, Table 1, Supplementary Table 2A).

### Associations between HAH severity and the pre-/post-exposure parameters

Among the pre-exposure parameters, only RET# was significantly associated with HAH severity (*r* = −0.315, *P* = 0.035). Among the post-exposure parameters, HAH severity was significantly and negatively associated with WBC (*r* = −0.319, *P* = 0.032), NEU# (*r* = −0.315, *P* = 0.035), and NEU% (*r* = −0.383, *P* = 0.009). Furthermore, HAH severity was positively associated with post-exposure LYM% (*r* = 0.348, *P* = 0.019). No other significant associations were observed between the remaining demographic or physiological parameters and HAH severity (all *P* > 0.05, Table 2, Supplementary Table 2B).

**Table 2:** Relationships between HAH severity score and all the variables.

	**Variables at sea level**		**Variables at 3,700 m**	
	**With HAH score *r***	***P*-value**	**With HAH score *R***	***P*-value**
**DEMOGRAPHIC DATA**				
Age (year)	0.276	0.066	The same as sea level	
BMI (kg/m^2^)	−0.017	0.914	The same as sea level	
**PHYSIOLOGICAL PARAMETERS**				
SBP (mmHg)	0.088	0.565	0.046	0.764
DBP (mmHg)	0.000	0.999	0.125	0.414
HR (beat/min)	−0.218	0.150	−0.278	0.064
SpO_2_ (%)	−0.151	0.323	0.145	0.341
**HEMATOLOGICAL PARAMETERS**				
WBC (10^9^/L)	−0.266	0.078	−**0.319**	<**0.05**
NEU# (10^9^/L)	−0.260	0.085	−**0.383**	<**0.01**
LYM# (10^9^/L)	−0.026	0.866	0.285	0.058
MON# (10^9^/L)	−0.190	0.210	−0.113	0.460
EOS# (10^9^/L)	−0.007	0.966	−0.038	0.805
NEU% (%)	−0.238	0.115	−**0.437**	<**0.01**
LYM% (%)	0.237	0.117	**0.348**	<**0.05**
MON% (%)	0.123	0.421	0.168	0.270
EOS% (%)	−0.055	0.720	0.040	0.793
RBC (10^12^/L)	−0.115	0.451	−0.252	0.096
HGB (g/L)	−0.066	0.665	−0.236	0.119
HCT (%)	0.036	0.813	−0.218	0.151
RET# (10^9^/L)	−**0.315**	<**0.05**	No data	
RET% (%)	−0.283	0.059	No data	

*HAH, High-altitude headache; BMI, body mass index; SBP, systolic blood pressure; DBP, diastolic blood pressure; HR, heart rate; SpO_2_, pulse oxygen saturation. The bold data represent significant*.

### Predictors and risk factors for HAH

Among the pre-exposure parameters, the univariate logistic regression analyses revealed that only RET# was significantly associated with HAH (*P* < 0.05, Table 3, Supplementary Table 2C). Among the post-exposure parameters, the univariate analyses revealed that HAH was significantly associated with WBC, NEU#, NEU%, and LYM% (all *P* < 0.05, Table 4, Supplementary Table 2C). The adjusted logistic regression analyses revealed that HAH was independently predicted by pre-exposure low RET#, and that low post-exposure NEU% was an independent risk factor for HAH (all *P* < 0.05, Table 5).

**Table 3:** Univariate logistic regression for each variable at sea level.

			**95% CI**		
**Risk factors**	**β-coefficient**	**Odds ratio**	**Lower**	**Upper**	***P*-value**
**DEMOGRAPHIC DATA**					
Age (year)	0.208	1.231	0.965	1.572	0.095
BMI (kg/m^2^)	−0.506	0.603	0.091	4.008	0.601
Smoking	0.199	1.220	0.626	2.378	0.560
Drinking	−0.043	0.958	0.699	1.313	0.789
**PHYSIOLOGICAL PARAMETERS**					
SBP (mmHg)	0.008	1.008	0.931	1.092	0.845
DBP (mmHg)	−0.006	0.994	0.905	1.092	0.903
HR (beat/min)	−0.037	0.964	0.905	1.028	0.261
SpO_2_ (%)	−0.527	0.590	0.249	1.401	0.232
**HEMATOLOGICAL PARAMETERS**					
WBC (10^9^/L)	−0.376	0.687	0.415	1.136	0.143
NEU# (10^9^/L)	−0.679	0.507	0.252	1.023	0.058
LYM# (10^9^/L)	−0.126	0.882	0.310	2.505	0.813
MON# (10^9^/L)	−3.168	0.042	0.000	19.531	0.312
EOS# (10^9^/L)	1.173	3.233	0.044	237.534	0.593
NEU% (%)	−0.064	0.938	0.867	1.015	0.111
LYM% (%)	0.057	1.058	0.977	1.146	0.167
MON% (%)	−0.082	0.921	0.736	1.153	0.473
EOS% (%)	0.964	2.623	0.016	443.888	0.713
RBC (10^12^/L)	−0.572	0.565	0.190	1.679	0.304
HGB (g/L)	−0.022	0.979	0.922	1.039	0.478
HCT (%)	−0.003	0.997	0.825	1.206	0.975
RET# (10^9^/L)	−**0.065**	**0.937**	**0.882**	**0.996**	<**0.05**
RET% (%)	−2.701	0.067	0.004	1.125	0.060

*CI, Confidence interval; BMI, body mass index; SBP, systolic blood pressure; DBP, diastolic blood pressure; HR, heart rate; SpO_2_, pulse oxygen saturation. The bold data represent significant*.

**Table 4:** Univariate logistic regression for each variable at 3,700 m.

			**95% CI**		
**Risk factors**	**β-coefficient**	**Odds ratio**	**Lower**	**Upper**	***P*-value**
**DEMOGRAPHIC DATA (THE SAME AS SEA LEVEL)**					
**Physiological parameters**					
SBP (mmHg)	−0.005	0.995	0.948	1.044	0.845
DBP (mmHg)	0.021	1.021	0.959	1.086	0.518
HR (beat/min)	−0.042	0.959	0.906	1.014	0.143
SpO_2_ (%)	0.167	1.182	0.952	1.468	0.130
**Hematological parameters**					
WBC (10^9^/L)	−**0.579**	**0.561**	**0.315**	**0.996**	<**0.05**
NEU# (10^9^/L)	−**0.961**	**0.382**	**0.177**	**0.825**	<**0.05**
LYM# (10^9^/L)	1.038	2.823	0.642	12.417	0.170
MON# (10^9^/L)	−1.445	0.236	0.002	22.793	0.536
EOS# (10^9^/L)	0.737	2.089	0.017	252.777	0.763
NEU% (%)	−**0.128**	**0.880**	**0.796**	**0.973**	<**0.05**
LYM% (%)	**0.126**	**1.134**	**1.020**	**1.261**	<**0.05**
MON% (%)	0.213	1.238	0.815	1.880	0.317
EOS% (%)	0.102	1.107	0.798	1.536	0.543
RBC (10^12^/L)	−0.907	0.404	0.096	1.697	0.216
HGB (g/L)	−0.036	0.964	0.912	1.020	0.206
HCT (%)	−0.103	0.902	0.730	1.114	0.338

*CI, Confidence interval; BMI, body mass index; SBP, systolic blood pressure; DBP, diastolic blood pressure; HR, heart rate; SpO_2_, pulse oxygen saturation. The bold data represent significant*.

**Table 5:** Adjusted logistic regression for HAH at sea level and 3,700 m.

			**95% CI**		
**Risk factors**	**β-coefficient**	**Odds ratio**	**Lower**	**Upper**	***P*-value**
**VARIABLES AT SEA LEVEL**					
RET# (10^9^/L)	−0.065	0.937	0.882	0.996	< 0.05
**VARIABLES AT 3,700 M**					
NEU% (%)	−0.128	0.880	0.796	0.973	<0.05

*CI, Confidence interval*.

## Discussion

The present study revealed that some hematological parameters may be novel independent risk factors/predictors for HAH. For example, HAH was associated with low pre-exposure values for RET#, RET%, and NEU#. In addition, the HAH group had low post-exposure values for NEU#, NEU%, and WBC, as well as high values for LYM%. Moreover, HAH was independently predicted by low pre-exposure RET#, while low post-exposure NEU% was an independent risk factor for HAH.

### HAH is associated with reticulocyte and neutrophil counts at sea level

The present study revealed that low pre-exposure RET# was associated with the development of HAH at an altitude of 3,700 m. Furthermore, the severity of HAH was negatively associated with RET#. In this context, reticulocytes are immature RBCs and circulate in the bloodstream for approximately 1–4 days before developing into young mature RBCs (Lombardi et al., 2013). Interestingly, hypoxia stress can stimulate the production of reticulocytes (MacNutt et al., 2013), and young mature RBCs have greater affinity for oxygen plus more efficient oxygen delivery to the tissues, compared to old RBCs (Samaja et al., 1991; Cohen and Matot, 2013). Moreover, a recent open-label randomized controlled trial revealed that erythropoietin (which stimulates hematopoiesis) is an effective prophylaxis for AMS (Heo et al., 2014). Therefore, our results may support the prevention of HAH using prophylactic induction of hematopoiesis in case with anticipated acute exposure to a hypoxic environment.

Our results also indicate that the HAH+ group had significantly lower pre-exposure NEU#, compared to the HAH− group. Previous studies have demonstrated that neutrophils undergo cellular adhesion and trans-endothelial migration from the blood stream during inflammation (Hall, 2015). In addition, hypobaric hypoxia could readily amplify pre-existing inflammation, which might lead to cerebral edema and AMS (Eltzschig and Carmeliet, 2011; Song et al., 2016). Furthermore, a recent study demonstrated that inflammation at sea level could predict the development of AMS (Lu et al., 2016). Thus, our results may indicate that inflammation at sea level is a risk factor for HAH.

### Neutrophil recruitment during HAH

Our results indicate that the HAH+ had significantly lower values for NEU# and NEU%, and higher values for LYM%. Furthermore, HAH severity was negatively associated with NEU# and NEU%, while HAH severity was positively associated with LYM%. Although the relevance of these findings is unclear, there are several potential explanations.

The first potential explanation is that neutrophils undergo trans-endothelial migration from the blood stream during inflammation, which is related to the similar mechanisms underlying stroke and AMS (Jin et al., 2010; Julian et al., 2011; Hall, 2015). Moreover, neutrophils are among the first cells to infiltrate the ischemic brain (within 30 min to a few hours after developing focal cerebral ischemia), where they amplify the inflammatory response with the help of cytokines (e.g., interleukin-1β [IL-1β], IL-6, and tumor necrosis factor-α [TNF-α]) that are produced by activated microglial cells (Ziemka-Nalecz et al., 2017). Intriguingly, our previous study revealed that plasma levels of IL-1β, IL-6, and TNF-α were positively correlated with AMS severity at 3,700 m, and another recent study revealed that plasma IL-6 levels were positively associated with AMS severity at 4,450 m and 5,129 m (Boos et al., 2016; Song et al., 2016). Thus, we speculate that hypobaric hypoxia induces brain injury by recruiting circulating neutrophils that aggravate neurogenic inflammation and lead to the development of HAH.

The second potential explanation is that neutrophil recruitment may be due to dampened anti-inflammatory IL-10 and upregulated C-C Motif Chemokine Ligand 8 (CCL8). For example, our recent transcriptome analysis revealed that IL-10 is downregulated in AMS, whereas CCL8 is upregulated (Liu et al., 2017). In addition, IL-10 is a general suppressor of the inflammatory response, while CCL8 promotes neutrophil recruitment (O'Boyle et al., 2007; Ouyang et al., 2011). Thus, we speculate that the lower NEU# and NEU% in patients with HAH are associated with the reduction of IL-10 and upregulation of CCL8, and this topic warrants urgent investigation. The third potential explanation is that the relatively high LYM% in patients with HAH could be the result of lower NEU%, as patients with and without HAH had similar values for LYM#.

### Independent predictors and risk factors for HAH

The adjusted logistic regression analyses revealed that low post-exposure NEU% was a risk factor for HAH. This may be because of the inflammatory response that is induced during HAH development. In addition, HAH was independently predicted by low RET#. This may reflect an impaired ability to produce young mature RBCs, which would decrease oxygen delivery to the tissues at high altitudes.

### Limitations

Ours is the first study to examine the hematological characteristics of HAH, and provides the first step toward linking the fields of hematology and HAH. However, the study also has several limitations. First, we only examined a small cohort of young Chinese men, which could have introduced age- or sex-related bias. Second, we did not have access to post-exposure reticulocyte data. Thus, our findings should be validated using larger and more heterogeneous cohorts, as well as more complete experimental data.

## Conclusion

The present study is the first to examine the hematological characteristics of HAH, and revealed that HAH was closely associated with WBC, NEU#, NEU%, LYM%, RET#, and RET%. In addition, HAH was independently predicted by low pre-exposure RET#, and low post-exposure NEU% was an independent risk factor for HAH.

## Author contributions

YG conceived and designed the study. BL and GW oversaw laboratory analyses and HH provided the overall supervision of the study. GX and BS did the statistical analysis or contributed the laboratory experiments. Both GW and BL contributed to sample and physiological data collections. HH drafted the report. All authors contributed to the interpretation of results, critical revision of the manuscript and approved the final manuscript. YG is the guarantor.

### Conflict of interest statement

The authors declare that the research was conducted in the absence of any commercial or financial relationships that could be construed as a potential conflict of interest.

## Abbreviations

AMS: Acute mountain sickness
CCL8: C-C Motif Chemokine Ligand 8
CI: confidence interval
DBP: diastolic blood pressure
HAH: high-altitude headache
HAH+: participants with HAH
HAH−: participants without HAH
HR: heart rate
IL-1β: interleukin-1β
IL-6: interleukin-6
IL-10: interleukin-10
SBP: systolic blood pressure
SpO_2_: pulse oxygen saturation
TNF-α: tumor necrosis factor-α.
