# Supplementary material for: Hematological Risk Factors for High-Altitude Headache in Chinese Men Following Acute Exposure at 3,700 m
Source: Front Physiol. 2017 Oct 17;8:801. doi: 10.3389/fphys.2017.00801 (PMC5651045; doi:10.3389/fphys.2017.00801)
Supplement: Supplementary file 1 [file Table1.DOCX]

Supplementary table 1. Abbreviation, Full Name and Unit of hematological parameters

| Abbreviation | Full Name | Unit |
| --- | --- | --- |
| WBC | white blood cell count | 10^9^/L |
| RBC | red blood cell count | 10^12^/L |
| HGB | hemoglobin | g/L |
| HCT | hematocrit | % |
| PLT | platelet count | 10^9^/L |
| MPV | mean platelet volume | fL |
| PCT | platelet crit | % |
| MCV | mean corpuscular volume | fL |
| MCH | mean corpuscular hemoglobin | pg |
| MCHC | the concentration of mean corpuscular hemoglobin | g/L |
| NEU% | differential percentages of neutrophil | % |
| LYM% | differential percentages of lymphocyte | % |
| MON% | differential percentages of monocyte | % |
| EOS%: | differential percentages of eosinophil | % |
| NEU# | differential counts of neutrophil | 10^9^/L |
| LYM# | differential counts of lymphocyte | 10^9^/L |
| MON# | differential counts of monocyte | 10^9^/L |
| EOS# | differential counts of eosinophil | 10^9^/L |
| RDW-SD | red cell distribution width | fL |
| RDW-CV | the coefficient of variation of red cell distribution width | % |
| PDW | [platelet](javascript:void(0);) [distribution](javascript:void(0);) [width](javascript:void(0);) | fL |
| P-LCR | platelet large cell ratio | % |
| RET% | the percentages of reticulocyte | % |
| RET# | reticulocyte count | 10^9^/L |
