# Supplementary material for: Hematological Risk Factors for High-Altitude Headache in Chinese Men Following Acute Exposure at 3,700 m
Source: Front Physiol. 2017 Oct 17;8:801. doi: 10.3389/fphys.2017.00801 (PMC5651045; doi:10.3389/fphys.2017.00801)
Supplement: Supplementary file 2 [file Table2.DOCX]

Supplementary table 2A. Differences of the remaining variables between HAH+ and HAH-

|  |  | Measurements at sea level | |  | Measurements at 3700 m | | |
| --- | --- | --- | --- | --- | --- | --- | --- |
|  |  | HAH+(23) | HAH-(22) |  | HAH+(23) | HAH-(22) |  |
| MPV (fL) |  | 10.60±0.54 | 10.30（1.28） |  | 11.35±0.62 | 11.04±0.84 |  |
| PCT (%) |  | 0.22±0.04 | 0.23±0.04 |  | 0.24±0.04 | 0.26±0.06 |  |
| MCV (fL) |  | 90.70（3.80） | 89.94±4.60 |  | 90.90（2.80） | 88.08±6.20 |  |
| MCH (pg) |  | 30.76（1.90） | 29.61±1.60 |  | 30.40（1.50） | 30.17±1.53 |  |
| MCHC (g/L) |  | 328.96±8.52 | 331.55±5.98 |  | 336.22±8.46 | 339.09±7.61 |  |
| RDW–SD (fL) |  | 41.55±1.65 | 41.55±1.95 |  | 42.60±1.76 | 42.00±1.70 |  |
| RDW–CV (%) |  | 12.80（0.70） | 12.90（0.63） |  | 13.10（1.00） | 13.10（0.63） |  |
| PDW (fL) |  | 12.47±1.37 | 12.10±1.77 |  | 13.73±1.30 | 13.10±1.91 |  |
| P-LCR (%) |  | 29.03±4.06 | 26.48±5.75 |  | 35.18±4.34 | 32.89±6.28 |  |
| PLT (109/L) |  | 207.87±31.01 | 228.95±52.78 |  | 208.22±34.49 | 233.91±60.00 |  |

HAH+: participants with high-altitude headache (HAH); HAH-: participants without HAH.

ALL variables represent insignificant.

Supplementary table 2B. Relationships between HAH severity and the remaining variables

|  | Variables at baseline | |  |  | Variables at 3700 m | | | |
| --- | --- | --- | --- | --- | --- | --- | --- | --- |
|  | With HAH score r | | *P* value |  | With HAH score R | | | *P* value |
| MPV (fL) | 0.174 |  | 0.252 |  | 0.188 |  | | 0.216 |
| PCT (%) | -0.112 |  | 0465 |  | -0.217 |  | | 0.152 |
| MCV (fL) | 0.182 |  | 0.231 |  | 0.244 |  | | 0.106 |
| MCH (pg) | 0.234 |  | 0.122 |  | 0.156 |  | | 0.306 |
| MCHC (g/L) | -0.078 |  | 0.610 |  | -0.181 |  | | 0.235 |
| RDW–SD (fL) | 0.013 |  | 0.933 |  | 0.135 |  | | 0.378 |
| RDW–CV (%) | -0.154 |  | 0.312 |  | 0.018 |  | | 0.906 |
| PDW (fL) | 0.136 |  | 0.371 |  | -0.215 |  | | 0.156 |
| P-LCR (%) | 0.222 |  | 0.143 |  | -0.185 | | 0.224 | |
| PLT (10^9^/L) | -0.213 |  | 0.159 |  | -0.258 | | 0.087 | |

HAH: high-altitude headache.

ALL variables represent insignificant.

Supplementary table 2C. Univariate logistic regression for the remaining variables at sea level and 3700 m

|  |  | | 95% CI | | |
| --- | --- | --- | --- | --- | --- |
| Risk factors | β-coefficient | Odds ratio | Lower | Upper | *P* value |
| **The remaining variables at sea level** | | | |  |  |
| MCV (fL) | 0.041 | 1.042 | 0.920 | 1.180 | 0.515 |
| MCH (pg) | 0.137 | 1.146 | 0.870 | 1.511 | 0.332 |
| MCHC (g/L) | -0.051 | 0.950 | 0.872 | 1.036 | 0.249 |
| RDW–SD (fL) | 0.000 | 1.000 | 0.718 | 1.394 | 0.998 |
| RDW–CV (%) | -0.001 | 0.999 | 0.976 | 1.023 | 0.962 |
| PDW (fL) | 0.159 | 1.172 | 0.798 | 1.723 | 0.418 |
| P-LCR (%) | 0.107 | 1.113 | 0.981 | 1.263 | 0.097 |
| PLT (10^9^/L) | -0.012 | 0.988 | 0.974 | 1.003 | 0.112 |
| **The remaining variables at 3700 m** | | | |  |  |
| MPV (fL) | 0.585 | 1.795 | 0.774 | 4.164 | 0.173 |
| PCT (%) | -8.387 | 0.000 | 0.000 | 71.982 | 0.194 |
| MCV (fL) | 0.074 | 1.077 | 0.958 | 1.210 | 0.215 |
| MCH (pg) | 0.065 | 1.067 | 0.766 | 1.487 | 0.702 |
| MCHC (g/L) | -0.047 | 0.954 | 0.883 | 1.031 | 0.237 |
| RDW–SD (fL) | 0.205 | 1.228 | 0.863 | 1.746 | 0.253 |
| RDW–CV (%) | -0.021 | 0.979 | 0.904 | 1.061 | 0.606 |
| PDW (fL) | 0.248 | 1.282 | 0.873 | 1.881 | 0.205 |
| P-LCR (%) | 0.082 | 1.086 | 0.967 | 1.219 | 0.162 |
| PLT (10^9^/L) | -0.011 | 0.989 | 0.976 | 1.002 | 0.091 |

CI: confidence interval.

ALL variables represent insignificant.

Employing independent t-test, Mann-Whitney U test, Spearman correlations analysis and univariate logistic regression, HAH exhibited insignificant associations with the remaining data (all *P* values > 0.05, **Supplementary table 2A, 2B and 2C**).
